# Genetic and Demographic Determinants of Fuchs Endothelial Corneal Dystrophy Risk and Severity

**DOI:** 10.1001/jamaophthalmol.2025.0109

**Published:** 2025-04-01

**Authors:** Siyin Liu, Amanda N. Sadan, Nihar Bhattacharyya, Christina Zarouchlioti, Anita Szabo, Marcos Abreu Costa, Nathaniel J. Hafford-Tear, Anne-Marie S. Kladny, Lubica Dudakova, Marc Ciosi, Ismail Moghul, Mark R. Wilkins, Bruce Allan, Pavlina Skalicka, Alison J. Hardcastle, Nikolas Pontikos, Catey Bunce, Darren G. Monckton, Kirithika Muthusamy, Petra Liskova, Stephen J. Tuft, Alice E. Davidson

**Affiliations:** 1 UCL Institute of Ophthalmology, London, UK; 2 Moorfields Eye Hospital, London, UK; 3 Eye Center, Faculty of Medicine, University of Freiburg, Freiburg, Germany; 4 Department of Paediatrics and Inherited Metabolic Disorders, First Faculty of Medicine, Charles University and General University Hospital in Prague, Prague, Czech Republic; 5 School of Molecular Biosciences, College of Medical, Veterinary and Life Sciences, University of Glasgow, Glasgow, UK; 6 Department of Ophthalmology, First Faculty of Medicine, Charles University and General University Hospital in Prague, Prague, Czech Republic; 7 National Institute for Health and Care Research (NIHR) BRC at the Royal Marsden NHS Foundation Trust and the Institute of Cancer Research, London, UK

**Keywords:** Fuchs endothelial corneal dystrophy, trinucleotide repeat expansion disease, age-related disorder, corneal dystrophy, CTG18.1, *TCF4*, *COL8A2*, *ZEB1*, *SLC4A11*, *AGBL1*, *LOXHD1*

## Abstract

**Importance:**

Understanding the pathogenic mechanisms of Fuchs endothelial corneal dystrophy (FECD) could contribute to developing gene-targeted therapies.

**Objective:**

To investigate associations between demographic data and age at first keratoplasty in a genetically refined FECD cohort.

**Design, Setting, and Participants:**

This retrospective cohort study recruited 894 individuals with FECD at Moorfields Eye Hospital (London) and General University Hospital (Prague). Ancestry was inferred from genome-wide SNP array data. CTG18.1 status was determined by short tandem repeat and/or triplet-primed PCR. One or more expanded alleles (≥50 repeats) were classified as expansion-positive (Exp+). Expansion-negative (Exp-) cases were exome sequenced.

**Main Outcome(s) and Measure(s):**

Association between variants in FECD-associated genes, demographic data and age at first keratoplasty.

**Results:**

Within the total cohort (n=894), 77.3% were Exp+. The majority of European (668/829, 80.6%) and South Asian (14/22, 63.6%) patients were Exp+. The percentage of females was higher (151, 74.4%) in the Exp- cohort compared to the Exp+ (395, 57.2%; difference = 17.2% [95% CI: 10.1% to 24.3%], *P*<.001). The median (IQR) age at first keratoplasty of the Exp+ patients (68.2 [63.2–73.6] years) was older than the Exp- patients (61.3 [52.6–70.4] years; difference = 6.5y [95% CI: 3.4y to 9.7y], *P*<.001). The CTG18.1 repeat length of the largest expanded allele within the Exp+ group was inversely correlated with the age at first keratoplasty (β = -0.087 [95% CI: -0.162 to -0.012], *P*=.02). The ratio of biallelic to monoallelic expanded alleles was higher in the FECD cohort (1:14) compared to an unaffected control group (1:94; *P*<.001), indicating that two Exp+ alleles were associated with increased disease penetrance compared with one expansion. We only identified potentially pathogenic variants (MAF <0.01; CADD >15) in FECD-associated genes in 13 (10.1%) Exp- individuals.

**Conclusions and Relevance:**

CTG18.1 expansions are present in most European and South Asian patients with FECD. CTG18.1 repeat length and zygosity status were associated with modifications in disease severity and penetrance. Known disease-associated genes account for only a minority of Exp- cases, with unknown risk factors associated with disease in the rest of this subgroup. These data may have implications for future FECD gene-targeted therapy development.

## Introduction

Fuchs endothelial corneal dystrophy (FECD) is a bilateral, progressive disease of the corneal endothelium that is a leading indication for keratoplasty in high-income countries.^1,2^ It is a genetically heterogeneous, variably penetrant, autosomal dominant trait. Most studies report a preponderance of females, with a ratio of 1.5 to 3.7 female per male.^3–9^ The disease appears to be more prevalent in European than East Asian or Middle Eastern populations.^10–14^ A recent FECD comorbidity association study demonstrated that female sex and European ancestry increase the risk of developing FECD by 4.6 fold and 5.5 fold, respectively.^15^ Depending on ancestry, 17% to 81% of FECD patients in these cohorts have one or more expanded copies of an intronic CTG repeat within the *TCF4* gene (termed CTG18.1; MIM: *602272.0007),^6–9,16–25^ making it, by far, the most common trinucleotide repeat expansion disease. Other rarer genetic causes have been identified through linkage analysis and candidate gene screening within familial cohorts.^26,27^ For example, heterozygous missense variants in *COL8A2* cause an early-onset and phenotypically distinct form of the disease.^28,29^ Rare variants in other genes, including *SLC4A11, ZEB1, AGBL1, LOXHD1* and *TCF4*, have also been associated with FECD, though several findings have not been replicated.^13,26,30–33^ In addition, three FECD genome-wide association studies (GWAS) have collectively identified twelve significant genomic loci but, excluding CTG18.1 expansion status, the causal risk variants driving these association signals remain elusive.^34–36^

Gene-targeted interventions to prevent or delay FECD progression are in development.^37–41^ However, their success will rely on identifying at-risk individuals before corneal endothelial function deteriorates and sight loss occurs. Here, we present an in-depth analysis of a large and extensively genotyped FECD cohort.

## Methods

### Participant recruitment

We recruited participants at Moorfields Eye Hospital (MEH), London and the General University Hospital (GUH), Prague, from September 2009 to July 2023, following the tenets of the Declaration of Helsinki. The study was approved by the Research Ethics Committees of University College London (UCL) (22/EE/0090), Moorfields Eye Hospital (MEH) London (13/LO/1084), or the General University Hospital (GUH) Prague (151/11 S-IV). All participants were diagnosed with FECD based on the documented finding of confluent corneal guttae seen by slit-lamp examination. They provided written informed consent and whole blood or saliva for DNA extraction. The potential effect of CTG18.1 genotype on phenotypic outcome was evaluated using two clinical parameters: A) age at recruitment (at date of whole blood or saliva sample collection); B) the age at first keratoplasty (at date of their first keratoplasty), which could be before or after recruitment. To prevent the confounding effect of traumatic endothelial cell loss, patients with a history of intraocular surgery, including cataract extraction, were excluded. Similarly, we only included patients who had a primary endothelial keratoplasty with or without phacoemulsification. This study followed Strengthening the Reporting of Observational Studies in Epidemiology guidelines for case-control studies, with workflow detailed in eFigure 1.

### DNA extraction and CTG18.1 genotyping

Genomic DNA was extracted from whole blood or saliva using a Gentra Puregene Blood kit (Qiagen) or Oragene saliva kit (Oragene OG-300, DNA Genotek). All samples were analyzed using a previously-described short tandem repeat (STR)-polymerase chain reaction (PCR) assay.^16,18,42^ Triplet repeat-primed (TP)-PCR was subsequently performed if only one CTG18.1 allele was detected, to determine if an allele longer than the STR-PCR detection maximum (∼125 repeats) was present. We defined cases with one or both alleles having ≥50 repeats as expansion-positive (Exp+) and those with biallelic alleles of <50 repeats as expansion-negative (Exp-).^17,42^

### Ancestry and relatedness

We genotyped all participants using a UK Biobank Axiom Array (Applied Biosystems). Genotypes were called using Axiom Analysis Suite software. Ancestry was inferred by principal component analysis (PCA) (FRAPOSA).^43^ We used 2,492 unrelated samples with known ancestry from the 1000 Genomes Project as a reference panel. Kinship analysis was performed using KING.^44^ Probands were defined as the first recruited individual within such kinships, and all cases identified as 2nd-degree cousins or more closely related to probands were excluded (kinship coefficient > 0.0884).

### Exome sequencing and rare variant analysis pipeline

Exome libraries were generated using a SureSelect Human All Exome V6 capture kit (Agilent) or a SeqCap EZ MedExome Enrichment Kit (Roche) and sequenced on either a HiSeq 4000 or 2500 platform (Illumina). Raw sequencing data were aligned using Burrows-Wheeler Aligner (BWA, 0.7.17).^45,46^ Variants and indels were called according to the Genome Analysis Toolkit Haplotypecaller (GATK, v4.4).^47^ Aligned data were interrogated for rare and potentially disease-associated coding variants in previously implicated in FECD genes: *COL8A2, ZEB1, SLC4A11, AGBL1, LOXHD1* and *TCF4*.^26,27,33^ Variants were annotated using Ensembl VEP (106.1),^48^ with the Combined Annotation Dependent Depletion (CADD, v1.6)^49^ and REVEL^50^ plugins. We defined variants of interest as having a CADD score >15 and a minor allele frequency (MAF) <0.01 in the Genome Aggregation Database (gnomAD, v3.1.2) in all genetic ancestry groups, excluding the Amish population.^51^ Variants of interest were verified by Sanger sequencing. SpliceAI was used to assess the effect of splice region variants on splicing.^52^

### Corneal endothelial transcriptome analysis

Cultured corneal endothelial cell (CEC) transcriptomes from four healthy control adults were queried to determine the relative abundance of the genes expressed within the corneal endothelium (EGAS50000000303).^33^ Briefly, FASTQ files were quantified with Salmon (GRCh38.p13, Ensembl v100, V1.4.0)^53^ and tximport (v.1.30.0)^54^ to generate normalized TPM gene-level counts. Genes with expression levels (TPM) ≤ 0.02 were considered not expressed within the corneal endothelium.

### Statistical analysis

Statistical analysis was performed using R (version 4.0.2, R Foundation for Statistical Computing). Data normality was assessed using Kolmogorov–Smirnov/Shapiro–Wilk tests. We used the χ^2^ test to compare categorical data. Wilcoxon signed-rank test was used to analyze non-parametric continuous variables. A linear regression model assessed the association between CTG18.1 repeat length of the largest expanded allele and age at first keratoplasty. Cases with repeat lengths of ≥125 repeats, which exceed the detection limit of STR-PCR, and cases with biallelic Exp+ where the repeat lengths of either allele could not be determined by the STR-PCR assay, were excluded from the regression analysis. Allele frequencies of European FECD cases and unaffected, aged (median 78.7y, interquartile range [IQR]: 73.9-82.7), European controls, as previously reported,^17^ were calculated to derive the observed and expected biallelic to monoallelic Exp+ allelic ratio, respectively. CTG18.1 was previously genotyped in this control group following the same genotyping approach applied by this study.^17^ The observed and expected ratios of monoallelic to biallelic Exp+ cases were compared. All *P* values were two-sided and no adjustments were applied for multiple analyses.

## Results

### FECD cohort sex and ancestry varied with CTG18.1 allelic distributions

**Figure 1:**
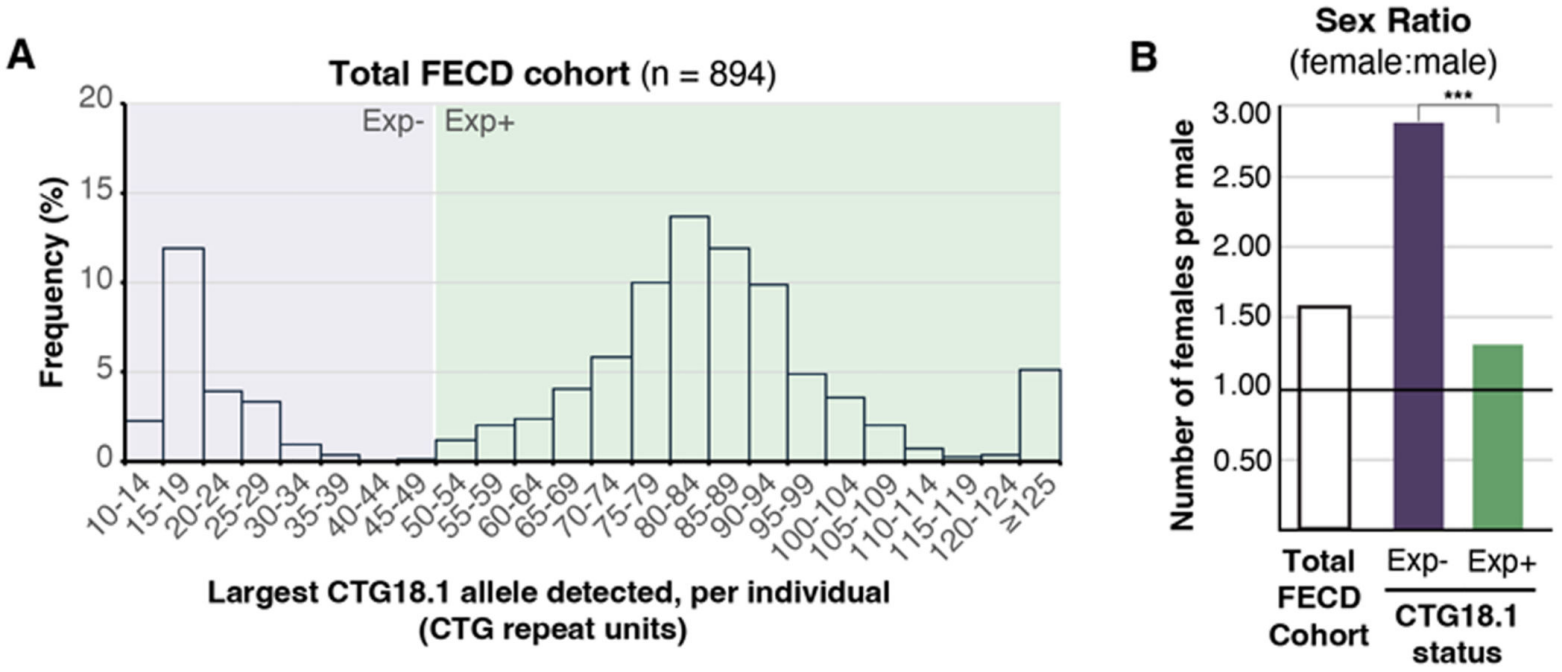
CTG18.1 repeat length is associated with FECD and its distribution varied with sex within a large FECD patient cohort. **(A)** Frequency histograms comparing the relative distribution of CTG18.1 repeat length within the total cohort (894). (**B**) Bar chart of the sex ratio across the total cohort (1.57 female:male, black bar) and subgroups stratified by CTG18.1 status: Exp- group (2.90 female:male, purple bar), Exp+ group (1.33 female:male, green bar). Abbreviation: CTG18.1 expansion positive allele defined as ≥50 CTG repeats; Exp-, CTG18.1 expansion negative allele defined as <50 CTG repeats.

We recruited 918 patients with FECD. Twenty-four were determined to be closely related and excluded, leaving 894 probands, of which 546 (61.1%) were females. PCA of genome-wide SNP array data showed that 829 (92.7%) were European (Table 1). CTG18.1 genotyping revealed that 691 (77.3%) participants had at least one expanded copy of the CTG18.1 allele, and 46 (5.1%) had bi-allelic expansions. More European patients had a CTG18.1 expansion (668, 80.6%) compared to non-Europeans (23, 35.4%; difference = 45.2% [95% CI: 33.3% to 57.1%], *P*<.001), in agreement with gnomAD (v3.1.2), which shows Europeans have the highest population frequency of CTG18.1 expansion.^55^ By comparing these data to the age and ethnicity-matched control cohort,^17^ harboring at least one expanded CTG18.1 allele conferred >78-fold risk for FECD in patients of European ancestry (odd ratio [OR] = 78.5; 95% CI: 50.3 to 122.6, *P*<.001). Overall, there was a lower proportion of Exp+ African cases (7, 18.9%) compared to European cases (difference = 61.7% [95% CI: 48.8% to 74.6%], *P*<.001), while the proportion of Exp+ cases between South Asian (14, 63.6%) and European groups was similar (difference = 17.0% (95% CI: -3.2% to 37.2%), *P*=0.09; Table 1; eTable 1-2; Figure 1A; eFigure 2).

The high proportion of females in the total FECD cohort (1.57 female-to-male ratio) validates numerous previous reports (Table 1; eTable 1). However, in the Exp- subgroup, 151 (74.4%) were female (2.90 ratio), which was higher than the Exp+ subgroup (395, 57.2%, 1.33 ratio; difference = 17.2% [95% CI: 10.1% to 24.3%], *P*<.001; Figure 1B).

### CTG18.1 repeat length and expanded allele dosage modified the age at first keratoplasty and disease penetrance, respectively

**Figure 2:**
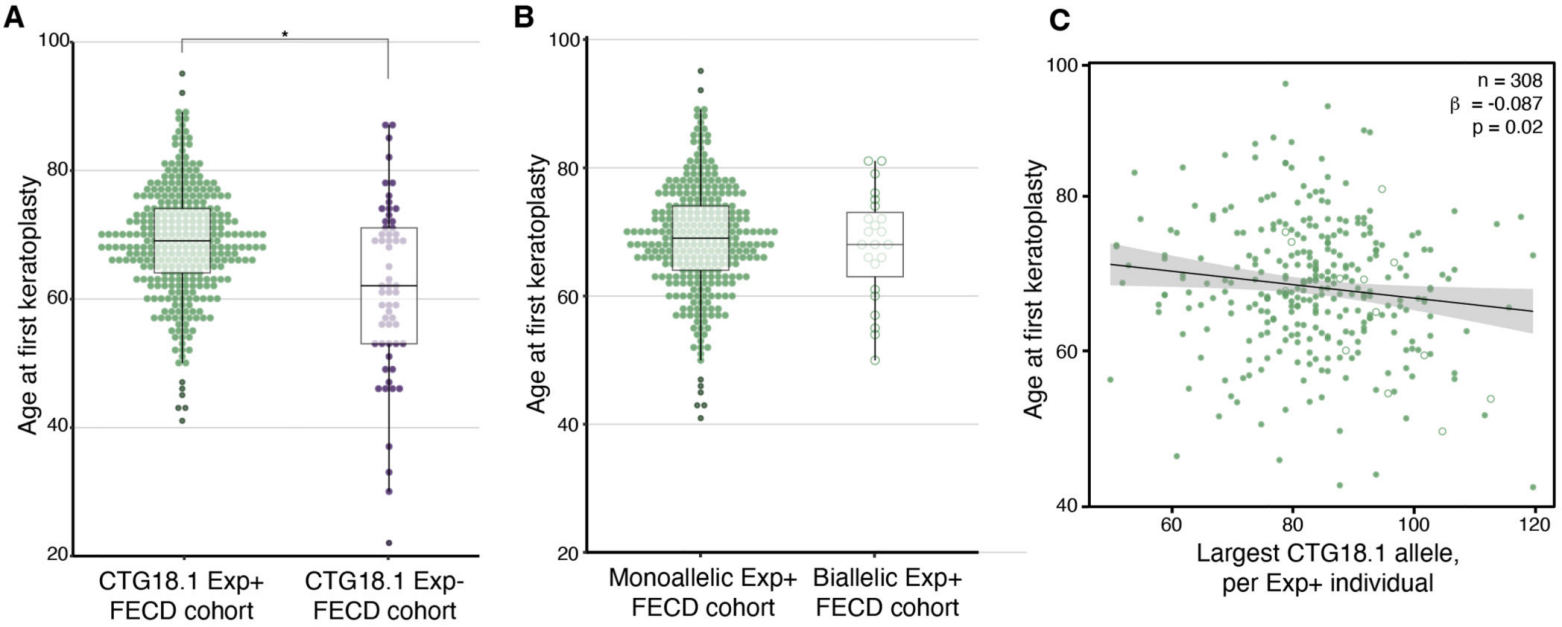
Age at first keratoplasty varies depending on CTG18.1 expansion status, repeat length and zygosity. (**A**) Age at first keratoplasty (median [IQR]) was more heterogeneous in the expansion-negative (Exp-) group (61.3y [52.6–70.4]) compared to the expansion-positive (Exp+) group (68.2y [63.2–73.6]), including both mono- and bi-allelic Exp+ cases (*P*<.001). (**B**)The median age at first keratoplasty in FECD patients with biallelic Exp+ (67.7y [62.4–72.4]) was not different (*P*<.69) than in monoallelic Exp+ patients (68.2y [63.3–73.7]).(**C**) The scatter plot demonstrates a negative correlation between the CTG18.1 repeat length and age at first keratoplasty in Exp+ patients (β = -0.087 [95% CI: -0.162 to -0.012], P = .02). *Green dots*, monoallelic Exp+ cases; *green open circles*, biallelic Exp+ cases.

The median (IQR) age at recruitment of the total FECD cohort was 69.7y (62.7–76.1), though the Exp- patients (66.7y [53.7–74.7]) were younger than Exp+ patients (70.2y [64.7–76.4]; Hodges-Lehmann-estimated difference [HL] = 4.7y [95% CI: 2.6y to 6.8y] *P*<.001; Table 2). Whilst both male and female Exp+ patients were recruited at a similar age, Exp- males tended to be recruited at a younger age than females (62.6y [50.8–73.6] vs 67.7y [55.8–75.1]; HL = 0.2y [95% CI: -1.3y to 1.6y], *P*=.13). After excluding cases that did not meet the inclusion criteria for genotype-keratoplasty data analysis, a higher proportion of Exp+ patients (382, 58.9%) had keratoplasty than the Exp- group (59, 34.9%; eTable 3). The median age at first keratoplasty for the Exp+ patients (68.2y [63.2–73.6]) was older than for the Exp- patients (61.3y [52.6–70.4]; HL = 6.5y [95% CI: 3.4y to 9.7y], *P*<.001; Table 2). This was likely due to a broader age distribution within Exp-, where there was a subset who had surgery at a relatively young age (Exp+ 40y–95y vs Exp- 22y–87y, *P*<.001) (Figure 2A).

The median age at first keratoplasty was similar for patients with biallelic CTG18.1 Exp+ (67.7y [62.4–72.4]) compared to those with monoallelic expansions (68.2y [63.3–73.7]; HL = 0.7y [95% CI: -2.8y to 4.5y], *P*<.69; Figure 2B; Table 2). Notably, the observed ratio of biallelic to monoallelic Exp+ cases, derived from homozygous to heterozygous Exp+ allelic ratios, was higher in the FECD cohort (1:14) compared to the expected ratio of Exp+ cases in an aged, unaffected group^17^ (1:94) (expected vs observed biallelic Exp+ cases: 7 vs 46; *P<.001*; eTable 4), suggesting that disease penetrance was higher in carriers of two expanded copies of CTG18.1.

Within the refined Exp+ patient group with sized CTG18.1 alleles (308/382), linear regression demonstrated a negative correlation between the CTG18.1 repeat length of the single largest expanded allele and age at first keratoplasty (β = -0.087 [95% CI: -0.162 to - 0.012], *P*=.02; eTable 5; Figure 2C).

### Rare coding variants in FECD-associated genes account for a minor fraction of missing heritability in Exp- cases

To explore the missing heritability in the Exp- group, exome data was generated for 128 Exp- patients. FECD-associated genes were interrogated for rare and potentially deleterious variants in conjunction with bulk CEC-specific RNAseq data. Analysis of the transcriptomic data revealed that neither *LOXHD1* nor *AGBL1* are expressed (eTable 6). This finding, in conjunction with the fact that neither gene has been replicated as FECD-associated,^26^ led us to discount variants in these genes. Within the remaining robustly validated gene set (*COL8A2, SLC4A11, ZEB1*, and *TCF4*), we only identified potentially disease-associated variants in 13 (10.1%) of 128 patients (Table 3).

Four Exp- patients had three qualifying heterozygous *COL8A2* missense variants (Table 3). Two harbored the same pathogenic missense variant, c.1363C>A p.(Gln455Lys), previously established to cause early-onset FECD (MIM #136800).^28^ Notably, both cases had corneal transplantation in their second or third decade (Table 3). Patients P1425 and P1726 harbored p.(Arg434His) and p.(Pro575Leu) variants, but without early-onset disease. P1425 had a keratoplasty at 69.5y, and P1726 was 62y when recruited and has not undergone surgery. Both variants have been associated with FECD, although lack of segregation with disease has been reported independently, suggesting they might be associated with incomplete penetrance or are non-causal.^28,57^

Two cases, P309 and P723, harbored heterozygous qualifying *SLC4A11* missense variants not previously associated with FECD. Interestingly, both variants alter the same amino acid residue: p.(Arg331Trp) and p.(Arg331Gln) (Table 3). In four cases, we identified qualifying *ZEB1* variants, including three heterozygous missense variants p.(Thr233Met), p.(Thr752Ala) and p.(Glu1033Asp) and a heterozygous splice region variant, c.794-7T>G, not predicted by SpliceAI to impact the splicing of any *ZEB1* transcripts (acceptor loss score Δ0.04) (Table 3). There were five qualifying *TCF4* variants in four previously reported patients,^33^ including three missense and two potentially loss-of-function variants (Table 3). In case P399, two consecutive missense and nonsense variants occurred *in cis*; c.[57G>T;58A>T]; p.[(Arg19Ser;Lys20*)]. For P723, the rare variant resulted in a synonymous change, c.66G>A p.(Glu22=), that is predicted to result in the loss of a native donor site (SpliceAI donor loss score Δ0.78).^33^

## Discussion

Genetic interrogation of this FECD cohort has revealed that i) dosage of expanded CTG18.1 alleles modifies penetrance, ii) variants in known associated genes only account for a minority of missing heritability in Exp- cases, iii) and the preponderance of female disease is associated with Exp- cases.

The prevalence of CTG18.1 expansions varied between ethnic groups, with a reported allele frequency of 2.95% in European, and 0.7%–1.8% in non-European populations.^55^ Here we also confirmed our previous finding^17^ that a single expanded allele conferred ≥78-fold increased risk of developing FECD. Thus the reported higher prevalence of FECD may be explained by the higher frequency of CTG18.1 expansions in the European population.^10–14^ We found that the proportion of South Asian patients (63.6%) with a CTG18.1 expansion was higher than in previous reports (17.3%-34.0%)^6,21^ which suggests that CTG18.1 expansion may also be a common driver of FECD in this population.

Studies have used a range of clinical metrics to examine the effect of CTG18.1 expansions on disease severity.^19,23,24,58^ In the majority of these studies, the history of cataract extraction and the type of keratoplasty performed were not considered. Our study aimed to examine the inherent biological link between genotype and phenotype, whilst making our best effort to control for potential external influences that could distort the relationship. Hence, strict exclusion criteria were applied for our genotype-phenotype analyses. Our data demonstrated that the length of the largest expanded allele inversely correlates with the age at first keratoplasty. However, the correlation was modest, suggesting that age at first keratoplasty may be too crude a surrogate marker of disease severity and that other genetic or environmental factors modify the phenotype. Future longitudinal studies involving early screening with genotyping will likely improve our understanding of the impact of CTG18.1 repeat length on FECD onset and progression. Repeat length is an established predictor of age at onset in some repeat-mediated diseases,^59,60^ but the correlation has been reported to be weak or absent in others.^61–65^ It is also possible that there may be a maximum repeat length threshold above which the phenotypic effect is constant.

In this study, DNA from blood/saliva was used to estimate the inherited allele length. We have previously shown that individuals with ≥50 CTG18.1 repeats detected in blood/saliva consistently display molecular hallmarks of repeat-mediated pathology in their CECs.^33,42^ However, it is important to recognise that expanded CTG18.1 alleles are consistently much larger in affected CECs due to somatic instability.^42^ Nonetheless, the inherited allele length estimates from stable cell populations (i.e. blood/saliva) are considered informative for genotype-phenotype correlations, as shown in previous studies of repeat-mediated disease.^66^

We observed a strong, approximately sevenfold, enrichment of biallelic expansion cases in our cohort, suggesting that two copies of the expanded repeat increase disease penetrance. However, patients with a biallelic CTG18.1 expansion did not have a younger age at first keratoplasty compared to those with a monoallelic expansion. Soliman et al. also found no differences in severity between these two groups when they compared clinical metrics such as Krachmer grade, central corneal thickness, and the proportion who had a keratoplasty.^23^ Thus, two copies of the expanded repeat appeared to increase disease penetrance without resulting in detectable signs of increased disease severity in the patient population.

Our data demonstrated that the Exp- group was more ethnically and phenotypically diverse. Despite 10% (13/128) of the exome-sequenced Exp- cases harboring rare qualifying variants in previously reported FECD genes, only one *COL8A2* variant (p.Gln455Lys) has previously been robustly demonstrated as an established cause of FECD.^26,28,67^ Additional analysis is required to validate all remaining variants reported here. Furthermore, future in-depth genomic interrogation will be required to identify other rare Mendelian causes and/or complex genetic risk factors of disease that may in-part explain the missing heritability in the Exp- subgroup, though we cannot exclude the possibility that some cases with the FECD phenotype will not have a genetic basis for their disease. The female preponderance in the overall FECD cohort suggest that sex-specific factors may contribute to FECD in some cases. For example, dysregulation of estrogen metabolite pathways in FECD CECs and sex-specific sensitivity to UV-induced mitochondrial damage *in vitro* and in animal models have been reported.^68–71^ It is plausible that behavioral^72^ and biological sex differences may have an additive effect, increasing disease risk in females within the Exp+ group, where genetic factors are the primary drivers. The pronounced female preponderance in the Exp- subgroup indicated that these sex-specific factors may play a more critical role in FECD cases in the absence of established genetic causes or risk factors.

### Limitations

The majority of patients included in this study are European, which should be noted when contextualizing conclusions regarding ancestry. Accurately determining the age of onset in FECD was impossible as the disease can be asymptomatic for many years. We, therefore, used two surrogates to estimate disease severity: the age at recruitment and the age at first keratoplasty in either eye. Both of these parameters are likely to be affected by uncontrolled variables such as referral practice and patient preference.

## Conclusions

Comprehensive genetic interrogation of this multi-centre FECD cohort provides novel insight into this heterogeneous disease, such as the effect of genotype on phenotypic outcomes. However, a proportion of cases remain genetically unsolved. Several novel CTG18.1-targeted interventions are in development, which may reduce the demand for corneal donor tissue.^17,37,38,40,41,73,74^ Our data indicates that CTG18.1 zygosity status and repeat length of the expanded allele should be considered for inclusion in the design of clinical trials. The success of any of these approaches depends upon population screening to identify individuals with CTG18.1 expansions before irreversible damage occurs. The higher prevalence of CTG18.1 expansions among European and South Asian patients means these populations may be particularly well-positioned to benefit from the development of CTG18.1-targeted therapies once integrated into clinical practices.

## Extended Data

**eFigure 1:**
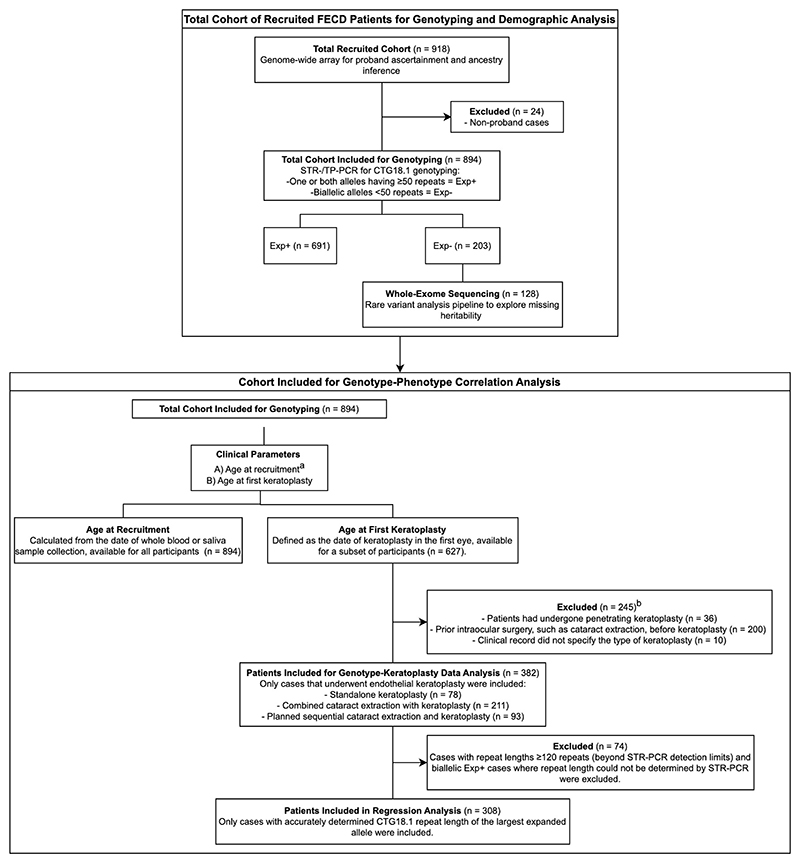
Overview of the study workflow. FECD, Fuchs endothelial corneal dystrophy; STR, short tandem repeat; TP, triplet repeat-primed; PCR, polymerase chain reaction; Exp+, CTG18.1 expansion positive allele defined as ≥50 CTG repeats; Exp-, CTG18.1 expansion positive allele defined as <50 CTG repeats. ^a^Recruitment may have occurred prior, concurrently or after keratoplasty; Not all participants had undergone keratoplasty. ^b^To control for non-genetic factors that could distort the genotype-phenotype relationship, only cases of endothelial keratoplasty were included, as thresholds for surgical intervention varied greatly in the previous era of penetrating keratoplasty compared to contemporary practices. Cases with prior intraocular surgeries (e.g., cataract extraction), that could potentially accelerate corneal endothelial cell loss, were also excluded.

**eFigure 2:**
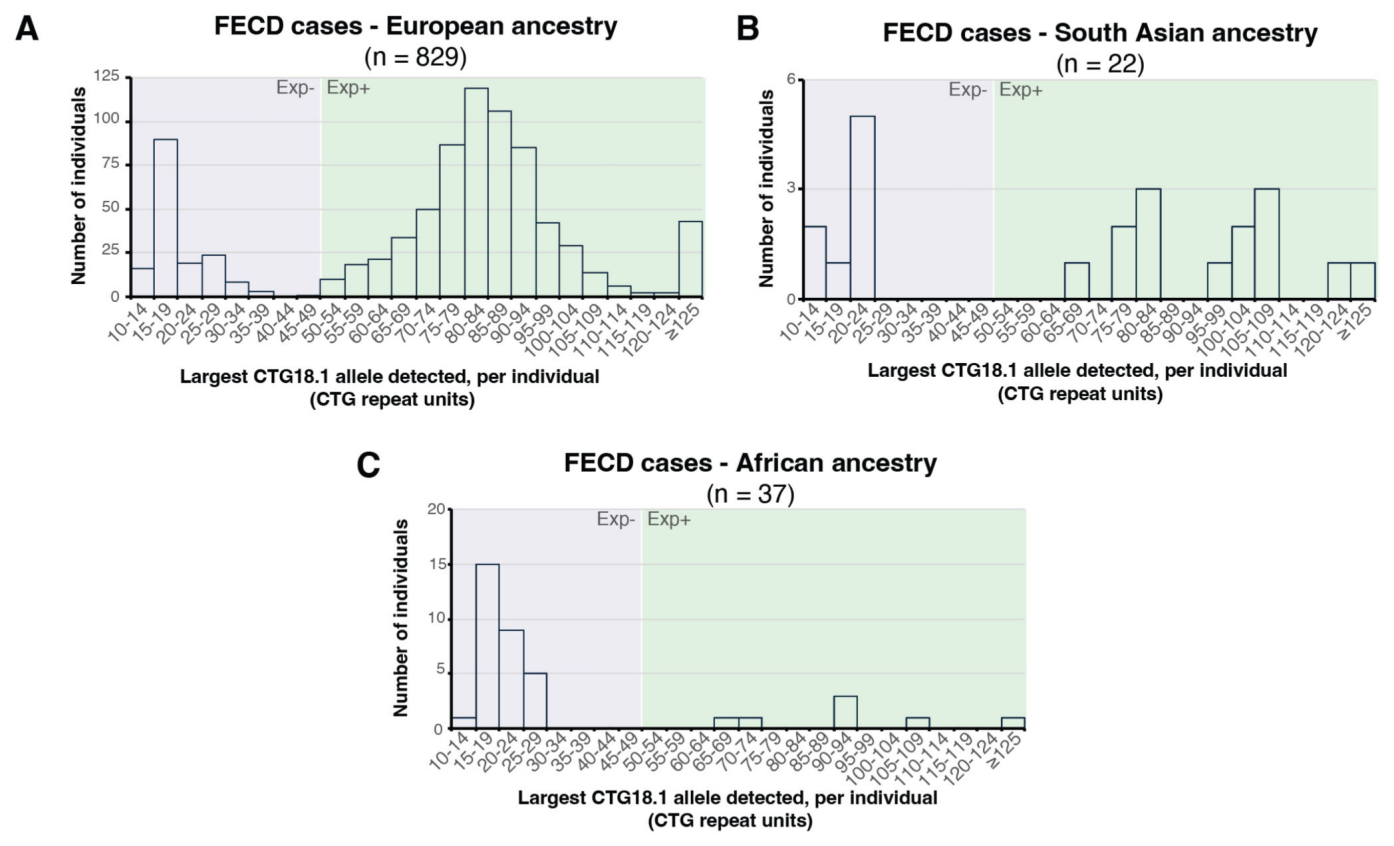
CTG18.1 repeat length distributions vary with ancestry within a large FECD patient cohort. Frequency histograms comparing the allelic distributions of CTG18.1 repeat length within (**A**) European (EUR; 829), (**B**) South Asian (SAS; 22) and (**C**) African (AFR; 37) ancestry groups. The percentage of European patients with a CTG18.1 expansion (80.7%) was greater than that of non-Europeans (35.4%). A larger proportion of South Asian patients also harbored at least one expanded CTG18.1 allele (63%), compared to patients of African ancestry (18%). Abbreviation: CTG18.1 expansion positive allele defined as ≥50 CTG repeats; Exp-, CTG18.1 expansion negative allele defined as <50 CTG repeats.

**eTable 1:** Summary of Sex Distribution and CTG18.1 Expansion Status in Fuchs Endothelial Corneal Dystrophy (FECD) Patient Cohort. Exp+, CTG18.1 expansion positive cases defined as one or both expanded alleles (≥50 CTG repeats); Exp-, CTG18.1 expansion negative cases defined as biallelic alleles of <50 repeats.

Sex	Number of cases	CTG18.1 Exp+			CTG18.1 Exp-
		Cases with ≥1 expanded allele	Monoallelic expanded cases	Biallelic expanded cases	
Females	546/894 (61.1%)	395/691 (57.2%)	371/645 (57.5%)	24/46 (52.2%)	151/203 (74.4%)
Males	348/894 (38.9%)	296/691 (42.8%)	274/645 (42.5%)	22/46 (47.8%)	52/203 (25.6%)

**eTable 2:** Summary of Recruitment Sites and CTG18.1 Expansion Status of Fuchs Endothelial Corneal Dystrophy (FECD) Patient Cohort. Exp+, CTG18.1 expansion positive cases defined as one or both expanded alleles (≥50 CTG repeats); Exp-, CTG18.1 expansion negative cases defined as biallelic alleles of <50 repeats; MEH, Moorfields Eye Hospital; GUH, General University Hospital.

Recruitment Site	Number of cases	CTG18.1 Exp+			CTG18.1 Exp-
		Cases with ≥1 expanded allele	Monoallelic expanded cases	Biallelic expanded cases	
MEH	563	430/563 (76.4%)	401/563 (71.2%)	29/563 (5.2%)	133/563 (23.6%)
GUH	331	262/331 (79.2%)	245/331 (74.0%)	17/331 (5.1%)	69/331 (20.8%)

**eTable 3:** Keratoplasty data of Fuchs endothelial corneal dystrophy (FECD) patient cohort stratified by sex and CTG18.1 genotype. Exp+, CTG18.1 expansion positive allele defined as ≥50 CTG repeats; Exp-, CTG18.1 expansion positive allele defined as <50 CTG repeats; MEH, Moorfields Eye Hospital; GUH, General University Hospital, IQR, interquartile range. ^a^Inclusion criteria: endothelial keratoplasty, whether as a standalone procedure, combined with phacoemulsification, or sequentially planned after phacoemulsification; Exclusion criteria: Penetrating keratoplasty, prior intraocular surgery, and unspecified keratoplasty type; the denominators include the total cohort, encompassing both operated and unoperated cases, after excluding those that meet the exclusion criteria.

	Number of cases	CTG18.1 Exp+			CTG18.1 Exp-
		Cases with ≥1 expanded allele	Monoallelic expanded cases	Biallelic expanded cases	
Cases Meeting Inclusion/Exclusion Criteria (N=649/894)^a^					
**Number of patients operated**	382/649 (58.9%)	323/480 (67.3%)	300/446 (67.3%)	23/34 (67.6%)	59/169 (34.9%)
Female	228/411 (55.5%)	181/283 (64.0%)	170/266 (63.9%)	11/17 (64.7%)	47/128 (36.7%)
Male	154/238 (64.7%)	142/197 (72.1%)	130/180 (72.2%)	12/17 (70.6%)	12/41 (29.3%)

**eTable 4:** Summary of CTG18.1 expansion status within Fuchs endothelial corneal dystrophy (FECD) probands of European ancestry and ethnicity-matched controls, where the derived allele frequency was used for expected and observed homozygous:heterzygous ratio calculation. Exp+, CTG18.1 expansion positive allele defined as ≥50 CTG repeats; Exp-, CTG18.1 expansion positive allele defined as <50 CTG repeats. ^a^Data presented from Zarouchlioti et al. 2018.^1 b^Control ratio under Hardy-Weinberg equilibrium that was confirmed in the control group (P=.617). ^c^Expected number of biallelic expanded cases based on control biallelic:monoallelic ratio and observed monoallelic expanded cases.

	Number of Individuals	CTG18.1 Exp-	CTG18.1 Exp+			Biallelic:monoallelic ratio	Expected biallelic expanded cases^c^
			Cases with ≥1 expanded allele	Monoallelic expanded cases	Biallelic Expanded cases		
FECD cases of European Ancestry	829	160	669	623	46	1:14	7
Ethnicity-matched Controls^a^	550	527	23	23	0	1:94^b^	<1

**eTable 5:** Linear regression models analyzing the relationship between CTG18.1 repeat length of the largest allele and age at first keratoplasty. Exp+, CTG18.1 expansion positive allele defined as ≥50 CTG repeats.

Model – single longest CTG18.1 allele measured per individual in all Exp+ patients	Age at first keratoplasty				
Variables	Number of cases	Adjusted R^2^	Regression coefficient	95% CI	*P* value
CTG18.1 repeat length of the largest allele	308	0.013	-0.087	-0.162 to -0.012	0.024

**eTable 6:** Summary of rare variants identified in *LOXHD1* and *AGBL1* from 128 FECD CTG18.1 Exp- probands analysed by exome sequencing. CEC, corneal endothelial cells; TPM, transcript per million; CADD, Combined Annotation Dependent Depletion; MAF, minor allele frequency; EUR, European; AFR, African American/African; FIN, Finnish; NFE, non-Finnish European, ASJ, Ashkenazi Jews; M, male; F, female; NS, no surgery; MEH, Moorfields Eye Hospital London; GUH, General University Hospital Prague. ^a^FRAPOSA predicted ancestry.

Gene CEC TPM	Transcript (ENST00000-)	Patient ID	Variant	CDS	Protein	CADD	gnomAD (v3.1.2)		Age at first Keratoplasty (Years)	Sex	Ancestry^a^	Family history	Site	Previously reported
							Total	Max (Population)						
*AGBL1* TPM = 0	614907.3	P824	chr15-86247772-A-T	c.628A>T	p.(Ile210Phe)	18.3	-	-	68	F	EUR	Unknown	MEH	No
		P371	chr15-86295271-T-C	c.2237T>C	p.(Leu746Pro)	25.2	-	-	71	F	EUR	Unknown	MEH	No
		P864	chr15-86674435-C-T	c.3157C>T	p.(Arg1053Trp)	36	0.001952 297/152158	0.008353 (ASJ) 29/3472	NS	M	EUR	Unknown	MEH	No
		P759	chr15-86907208-C-T	c.3280C>T	p.(Arg1094Trp)	16.8	0.001007 153/152002	0.003020 (AFR) 125/41394	45	F	AFR	Unknown	MEH	No
*LOXHD1* TPM = 0.02	642948.1	P2185	chr18-46610832-T-G	c.703A>C	p.(Lys235Gln)	26.0	0.00000656 1/152220	0.0000147 1/68030	NS	M	EUR	No	GUH	No
		P327	chr18-46592017-G-A	c.1570C>T	p.(Arg524Cys)	30	0.002833 431/152158	0.008903 (AMR) 136/15276	68	F	EUR	Unknown	MEH	No
		P354	chr18-46592017-G-A	c.1570C>T	p.(Arg524Cys)	30	0.002833 431/152158	0.008903 (AMR) 136/15276	73	F	EUR	Unknown	MEH	No
		P722	chr18-46541815-G-A	c.3874C>T	p.(Leu1292Phe)	25.7	0.0003088 47/152222	0.001223 (FIN) 13/10626	74	M	EUR	Unknown	MEH	No
		P2180	chr18-46541787-A-C	c.3902T>G	p.(Leu1301Arg)	16.2	0.00000657 1/152206	0.00006542 (AMR) 1/15286	NS	F	EUR	No	GUH	No
		P759	chr18-46533293-C-T	c.4244G>A	p.(Arg1415Gln)	27.3	0.0006768 103/152190	0.002293 (AFR) 95/41436	45	F	AFR	Unknown	MEH	No
		P523	chr18-46529227-G-A	c.4480C>T	p.(Arg1494Ter)	42	0.0006249 95/152026	0.001152 (ASJ) 4/3472	74	F	EUR	Unknown	MEH	No
		P399	chr18-46522163-G-A	c.5023C>T	p.(Arg1675Cys)	19.2	0.001512 230/152102	0.006743 (AMR) 103/15276	57	F	AFR	Unknown	MEH	No
		P788	chr18-46505914-G-T	c.5802C>A	p.(Asn1934Lys)	21.3	0.003082 469/152198	0.005624 (AMR) 86/15292	77	F	EUR	Unknown	MEH	No
		P522	chr18-46477568-G-A	c.6726C>T	p.(Thr2242Thr)	17.6	0.00002627 4/152258	0.00004823 (AFR) 2/41472	66	F	EUR	Unknown	MEH	No
		P737	chr18-46477553-G-A	c.6741C>T	p.(Ala2247Ala)	18.9	0.003061 466/152228	0.00463 (NFE) 315/68032	76	F	NA	Unknown	MEH	No

## Supplementary Material

eFigure

## Figures and Tables

**Table 1:** Summary of Fuchs endothelial corneal dystrophy (FECD) patient cohort demographics and CTG18.1 expansion status. Exp+, CTG18.1 expansion positive cases defined as one or both expanded alleles (≥50 CTG repeats); Exp-, CTG18.1 expansion negative cases defined as biallelic alleles of <50 repeats. The cohort data presented has been updated from an earlier publication.^17^ ^a^ Determined by principal component analysis using FRAPOSA.^43^

	Number of cases	CTG18.1 Exp+			CTG18.1 Exp-
		Cases with ≥1 expanded allele	Monoallelic expanded cases	Biallelic expanded cases	
**Genotyped patients**	894	691 (77.3%)	645 (72.1%)	46 (5.1%)	203 (22.7%)
**(Female:Male Ratio)**	1.57 (546:348)	1.33 (395:296)	1.35 (371:274)	1.09 (24:22)	2.90 (151:52)
**Ancestry^a^**					
**European**	829/894 (92.7%)	668/829 (80.6%)	622/829 (75.0%)	46/829 (5.5%)	161/829 (19.4%)
**African**	37/894 (4.1%)	7/37 (18.9%)	7/37 (18.9%)	0/37 (0.0%)	30/37 (81.1%)
**South Asian**	22/894 (2.5%)	14/22 (63.6%)	14/22 (63.6%)	0/22 (0.0%)	8/22 (36.4%)
**East Asian**	3/894 (0.3%)	1/3 (33.3%)	1/3 (33.3%)	0/3 (0.0%)	2/3 (66.7%)
**American Admixture**	3/894 (0.3%)	1/3 (33.3%)	1/3 (33.3%)	0/3 (0.0%)	2/3 (66.7%)

**Table 2:** Age at recruitment and first keratoplasty of Fuchs endothelial corneal dystrophy (FECD) patient cohort stratified by sex and CTG18.1 genotype. Exp+, CTG18.1 expansion positive allele defined as ≥50 CTG repeats; Exp-, CTG18.1 expansion positive allele defined as <50 CTG repeats; IQR, interquartile range. ^a^Inclusion criteria: endothelial keratoplasty, whether as a standalone procedure, combined with phacoemulsification, or sequentially planned after phacoemulsification; Exclusion criteria: Penetrating keratoplasty, prior intraocular surgery, and unspecified keratoplasty type; the denominators include the total cohort, encompassing both operated and unoperated cases, after excluding those that meet the exclusion criteria.

	Age (years)	CTG18.1 Exp+			CTG18.1 Exp-
		Cases with ≥1 expanded allele	Monoallelic expanded cases	Biallelic expanded cases	
**Median age at recruitment (IQR; in years)**					
**Total genotyped cohort (N=894)**	69.7y (62.7–76.1)	70.2y (64.7–76.4)	70.3y (64.7–76.5)	69.4y (64.9–73.1)	66.7y (53.7–74.7)
Female	69.6y (62.7–76.4)	70.2y (64.5–77.0)	70.1y (64.5–77.1)	71.1y (64.3–75.6)	67.7y (55.8–75.1)
Male	69.9y (62.8–75.5)	70.4y (64.9–75.9)	70.6y (64.8–76.1)	68.7y (65.44–71.8)	62.6y (50.8–73.6)
**Median age at first keratoplasty (IQR; in years)**					
**Cases Meeting** **Inclusion/Exclusion Criteria** **(N=649/894)^a^**	67.8y (61.8–73.1)	68.2y (63.2–73.6)	68.2y (63.3–73.7)	67.7y (62.4–72.4)	61.3y (52.6–70.4)
Female (N=228)	67.4y (61.5–72.8)	67.6y (63.0–72.9)	67.5y (63.1–72.9)	69.4y (62.4–73.5)	62.6y (54.0–71.0)
Male (N=154)	69.1y (62.1–73.5)	69.3y (63.6–74.0)	69.4y (63.6–75.0)	67.6y (64.1–71.2)	57.1y (51.6–63.4)

**Table 3:** Summary of rare and potentially pathogenic variants identified in FECD-associated genes from 128 FECD CTG18.1 Exp- probands analysed by exome sequencing. Relatives were not included in the main analysis even though identified here. Variants were classified according to the guidelines of the American College of Medical Genetics and Genomics/Association for Molecular Pathology (ACMG/AMP), utilising Franklin by Genoox platform alongside manual evaluation. CEC, corneal endothelial cells; TPM, transcript per million; CADD, Combined Annotation Dependent Depletion; MAF, minor allele frequency; EUR, European; AFR, African American/African; FIN, Finnish; NFE, non-Finnish European, ASJ, Ashkenazi Jews; M, male; F, female; NS, no surgery; MEH, Moorfields Eye Hospital London; GUH, General University Hospital Prague. ^a^FRAPOSA predicted ancestry.

T3Gene CEC TPM	Transcript (ENST00000-)	Patient ID	Variant	CDS	Protein	CADD	gnomAD (v3.1.2)		Age at first Keratoplasty (Years)	Sex	Ancestry^a^	Family history	Site	Previously reported	ACMG/AMP classification
							Total	Max (Population)							
*COL8A2* TPM = 806.3	397799.2	P1425	chr1-36098380-C-T	c.1301G>A	p.(Arg434His)	20.6	0.0008958 136/151828	0.001452 (SAS) 4/4820	69	M	EUR	No	GUH	56	Benign
		P573	chr1-36098318-G-T	c.1363C>A	p.(Gln455Lys)	19.2	-	-	22	F	EUR	Father	MEH	28	Pathogenic
		P836	chr1-36098318-G-T	c.1363C>A	p.(Gln455Lys)	19.2	-	-	38	F	EUR	No	MEH	28	Pathogenic
		P1726	chr1-36097957-G-A	c.1724C>T	p.(Pro575Leu)	24.7	0.001399 213/152202	0.003952 (FIN) 42/10628	NS	M	EUR	No	GUH	28	Benign
*ZEB1* TPM = 17.2	424869.6	P312	chr10-31514613-C-T	c.698C>T	p.(Thr233Met)	26.1	0.0000263 4/152066	0.0006219 (SAS) 3/4824	78	F	SAS	No	MEH	No	VUS
		P1812	chr10-31520119-T-G	c.794-7T>G	-	16.6	-	-	NS	F	EUR	No	GUH	No	VUS
		P733	chr10-31521586-A-G	c.2254A>G	p.(Thr752Ala)	17.6	0.001274 194/152228	0.001969 (NFE) 134/68042	46	F	EUR	No	MEH	No	Benign
		P469	chr10-31526985-G-C	c.3099G>C	p.(Glu1033Asp)	17.6	0.000006576 1/152076	0.0000147 (NFE) 1/68032	87	M	EUR	No	MEH	No	VUS
*TCF4* TPM = 203.7	354452.8	P1400	chr18-55261512-G-A	c.944C>T	p.(Ala315Val)	26.3	0.0006243 95/152160	0.001029 (NFE) 70/68028	NS	F	EUR	Brother	GUH	33	Benign
	544241.6	P351	chr18-55403689-A-G	c.26T>C	p.(Ile9Thr)	18.4	0.000006576 1/152076	0.0000147 (NFE) 1/68032	75	F	EUR	Yes - first degree	MEH	33	VUS
	566286.5	P723	chr18-55588470-C-T	c.66G>A	p.(Glu22=)	20.0	0.002131 324/152022	0.007271 (AFR) 301/41398	35	M	AFR	Unknown	MEH	33	VUS
		P399	chr18-55588478-T-A	c.58A>T	p.(Lys20Ter)	18.8	-	-	57	F	AFR	Unknown	MEH	33	VUS
		P399	chr18-55588479-C-A	c.57G>T	p.(Arg19Ser)	17.4	-	-	57	F	AFR	Unknown	MEH	33	VUS
*SLC4A11* TPM = 2396.4	642402.1	P309	chr20-3231200-G-A	c.991C>T	p.(Arg331Trp)	16.6	0.001308 199/152192	0.002305 (ASJ) 8/3470	70	F	EUR	No	MEH	No	Benign
		P723	chr20-3231199-C-T	c.992G>A	p.(Arg331Gln)	17.8	0.001538 234/152168	0.005477 (AFR) 227/41446	35	M	AFR	Unknown	MEH	No	Benign

## Data Availability

In this study we used patient identifiable information. For this reason raw data can not be made publicly available due to confidentiality considerations.

## Key Points

### Question

How are demographics and genetic risk factors associated with FECD disease risk and severity?

### Findings

This multi-center FECD cohort (n=894) study reveals *TCF4* repeat expansions (CTG18.1) were associated with disease in 77.3% of total cases, with longer repeats and biallelic expansions correlating with earlier keratoplasty and increased penetrance, respectively. Female overrepresentation was most pronounced in cases without CTG18.1 expansions, where missing heritability remained high.

### Meaning

Demographic factors and molecular diagnosis, including CTG18.1 repeat length and expanded allele dosage, are metrics that might inform future therapeutic strategies and clinical trial design.
