# Supplementary material for: Genetic and Demographic Determinants of Fuchs Endothelial Corneal Dystrophy Risk and Severity
Source: JAMA Ophthalmol. Author manuscript; Available in PMC 2025 Mar 19. (PMC11907363; doi:10.1001/jamaophthalmol.2025.0109)
Supplement: eFigure [file EMS203303-supplement-eFigure.docx]

**Online-Only Supplements**

**eFigure 1. Overview of the study workflow.**

**eFigure 2.** **CTG18.1 repeat length distributions vary with ancestry within a large FECD patient cohort.**

**eTable 1. Summary of Sex Distribution and CTG18.1 Expansion Status in Fuchs Endothelial Corneal Dystrophy (FECD) Patient Cohort.**

**eTable 2. Summary of Recruitment Sites and CTG18.1 Expansion Status of Fuchs Endothelial Corneal Dystrophy (FECD) Patient Cohort.**

**eTable 3. Keratoplasty data of Fuchs endothelial corneal dystrophy (FECD) patient cohort stratified by sex and CTG18.1 genotype.**

**eTable 4. Summary of CTG18.1 expansion status within Fuchs endothelial corneal dystrophy (FECD) probands of European ancestry and ethnicity-matched controls, where the derived allele frequency was used for expected and observed homozygous:heterzygous ratio calculation.**

**eTable 5. Linear regression models analyzing the relationship between CTG18.1 repeat length of the largest allele and age at first keratoplasty.**

**eTable 6. Summary of rare variants identified in *LOXHD1* and *AGBL1* from 128 FECD CTG18.1 Exp- probands analysed by exome sequencing.**

**Online-Only References**

**Online-Only Figure**

**
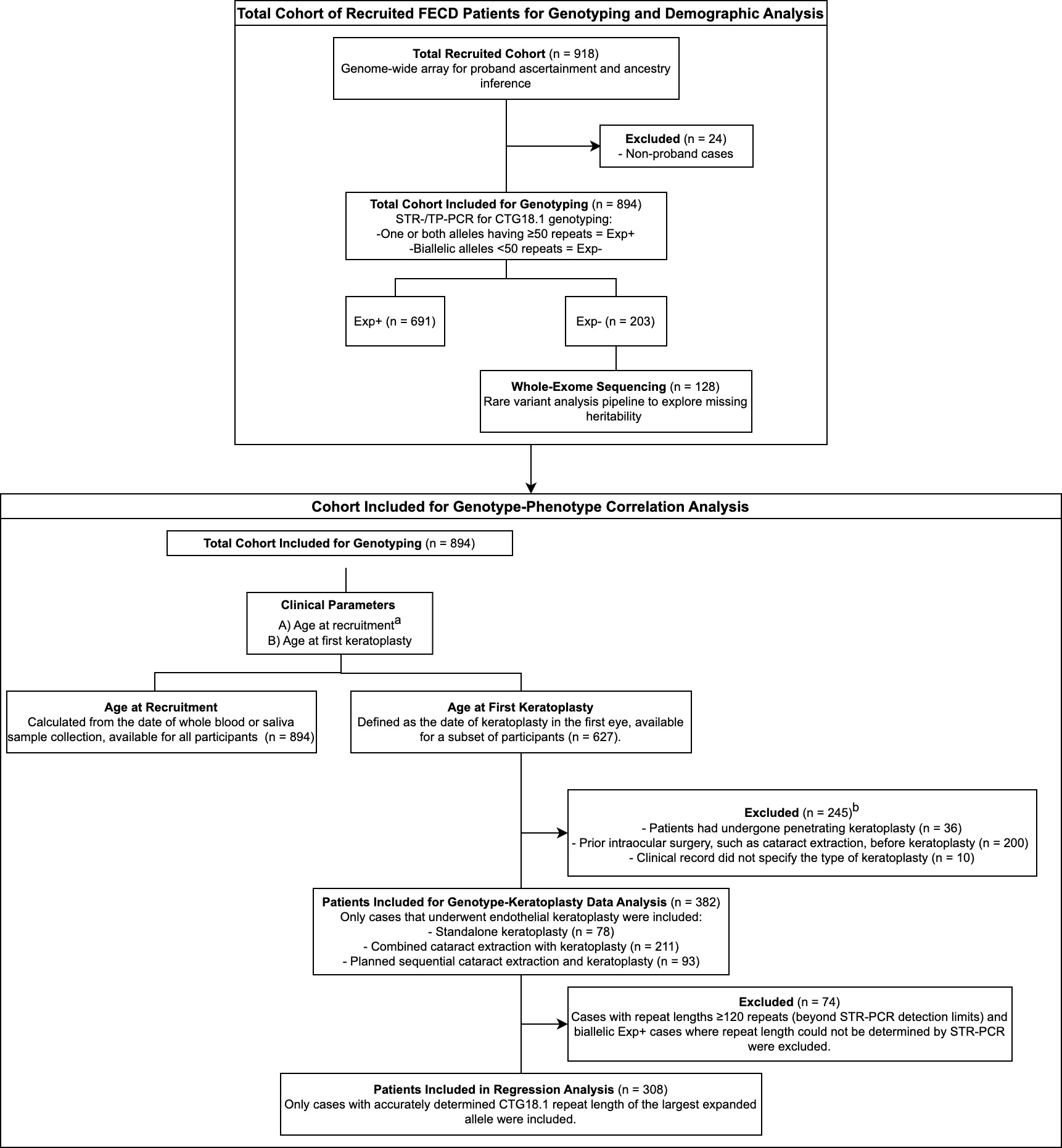
**

**eFigure 1. Overview of the study workflow.** FECD, Fuchs endothelial corneal dystrophy; STR, short tandem repeat; TP, triplet repeat-primed; PCR, polymerase chain reaction; Exp+, CTG18.1 expansion positive allele defined as ≥50 CTG repeats; Exp-, CTG18.1 expansion positive allele defined as <50 CTG repeats. ^a^Recruitment may have occurred prior, concurrently or after keratoplasty; Not all participants had undergone keratoplasty. ^b^To control for non-genetic factors that could distort the genotype-phenotype relationship, only cases of endothelial keratoplasty were included, as thresholds for surgical intervention varied greatly in the previous era of penetrating keratoplasty compared to contemporary practices. Cases with prior intraocular surgeries (e.g., cataract extraction), that could potentially accelerate corneal endothelial cell loss, were also excluded.

**
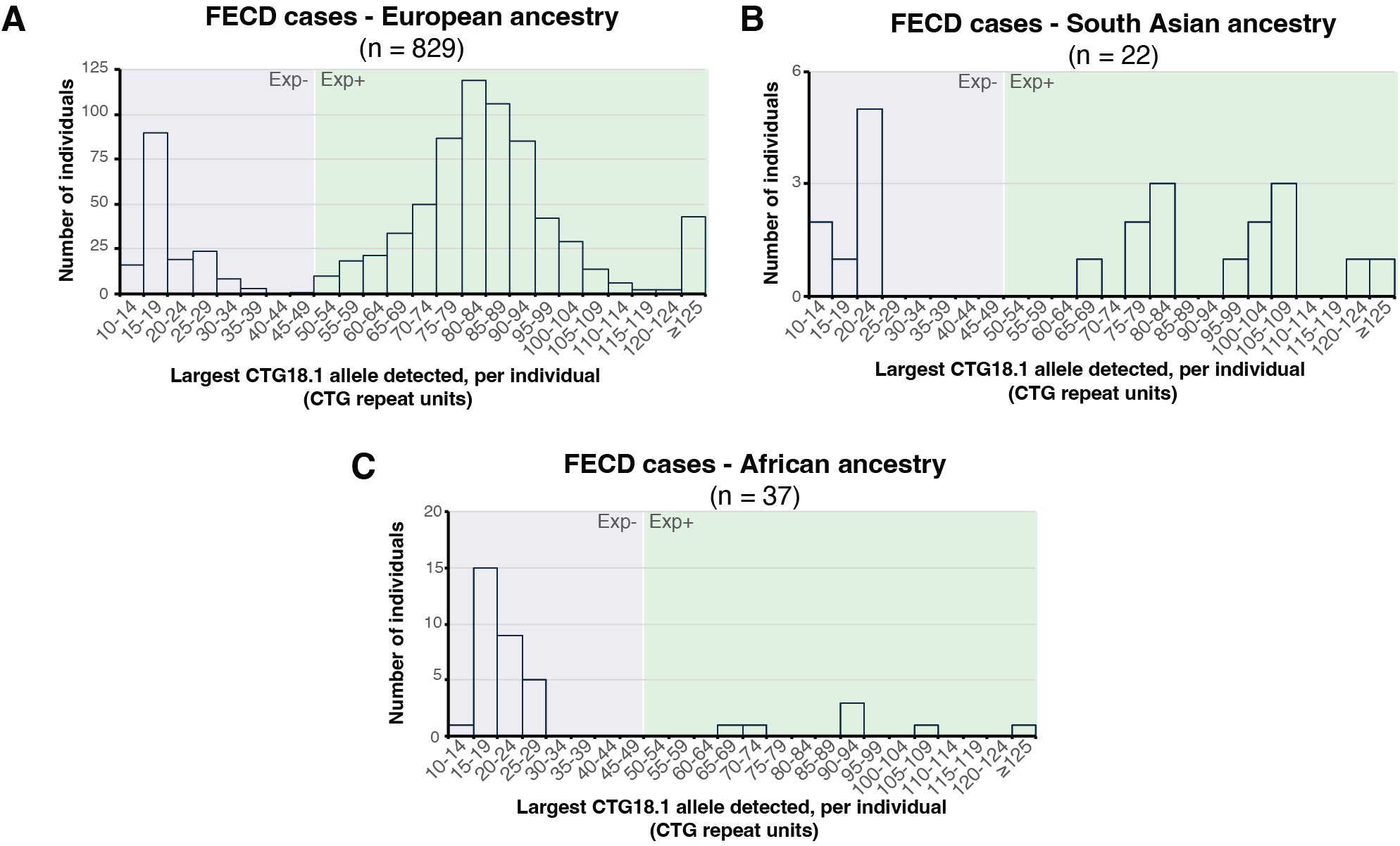
**

**eFigure 2.** **CTG18.1 repeat length distributions vary with ancestry within a large FECD patient cohort.** Frequency histograms comparing the allelic distributions of CTG18.1 repeat length within (**A**) European (EUR; 829), (**B**) South Asian (SAS; 22) and (**C**) African (AFR; 37) ancestry groups. The percentage of European patients with a CTG18.1 expansion (80.7%) was greater than that of non-Europeans (35.4%). A larger proportion of South Asian patients also harbored at least one expanded CTG18.1 allele (63%), compared to patients of African ancestry (18%). Abbreviation: CTG18.1 expansion positive allele defined as ≥50 CTG repeats; Exp-, CTG18.1 expansion negative allele defined as <50 CTG repeats.

**Online-Only Tables**

| **Sex** | **Number of cases** | **CTG18.1 Exp+** | | | **CTG18.1 Exp-** |
| --- | --- | --- | --- | --- | --- |
|  |  | **Cases with ≥1 expanded allele** | **Monoallelic expanded cases** | **Biallelic expanded cases** |  |
| Females | 546/894 (61.1%) | 395/691 (57.2%) | 371/645 (57.5%) | 24/46 (52.2%) | 151/203 (74.4%) |
| Males | 348/894 (38.9%) | 296/691 (42.8%) | 274/645 (42.5%) | 22/46 (47.8%) | 52/203 (25.6%) |

**eTable 1. Summary of Sex Distribution and CTG18.1 Expansion Status in Fuchs Endothelial Corneal Dystrophy (FECD) Patient Cohort.** Exp+, CTG18.1 expansion positive cases defined as one or both expanded alleles (≥50 CTG repeats); Exp-, CTG18.1 expansion negative cases defined as biallelic alleles of <50 repeats.

| **Recruitment Site** | **Number of cases** | **CTG18.1 Exp+** | | | **CTG18.1 Exp-** |
| --- | --- | --- | --- | --- | --- |
|  |  | **Cases with ≥1 expanded allele** | **Monoallelic expanded cases** | **Biallelic expanded cases** |  |
| MEH | 563 | 430/563 (76.4%) | 401/563 (71.2%) | 29/563 (5.2%) | 133/563 (23.6%) |
| GUH | 331 | 262/331 (79.2%) | 245/331 (74.0%) | 17/331 (5.1%) | 69/331 (20.8%) |

**eTable 2. Summary of Recruitment Sites and CTG18.1 Expansion Status of Fuchs Endothelial Corneal Dystrophy (FECD) Patient Cohort.** Exp+, CTG18.1 expansion positive cases defined as one or both expanded alleles (≥50 CTG repeats); Exp-, CTG18.1 expansion negative cases defined as biallelic alleles of <50 repeats; MEH, Moorfields Eye Hospital; GUH, General University Hospital.

|  | **Number of cases** | **CTG18.1 Exp+** | | | **CTG18.1 Exp-** |
| --- | --- | --- | --- | --- | --- |
|  |  | **Cases with ≥1 expanded allele** | **Monoallelic expanded cases** | **Biallelic expanded cases** |  |
| Cases Meeting Inclusion/Exclusion Criteria (N=649/894)^a^ | | | | | |
| **Number of patients operated** | 382/649 (58.9%) | 323/480 (67.3%) | 300/446 (67.3%) | 23/34 (67.6%) | 59/169 (34.9%) |
| Female | 228/411 (55.5%) | 181/283 (64.0%) | 170/266 (63.9%) | 11/17 (64.7%) | 47/128 (36.7%) |
| Male | 154/238 (64.7%) | 142/197 (72.1%) | 130/180 (72.2%) | 12/17 (70.6%) | 12/41 (29.3%) |

**eTable 3. Keratoplasty data of Fuchs endothelial corneal dystrophy (FECD) patient cohort stratified by sex and CTG18.1 genotype.** Exp+, CTG18.1 expansion positive allele defined as ≥50 CTG repeats; Exp-, CTG18.1 expansion positive allele defined as <50 CTG repeats; MEH, Moorfields Eye Hospital; GUH, General University Hospital, IQR, interquartile range. ^a^Inclusion criteria: endothelial keratoplasty, whether as a standalone procedure, combined with phacoemulsification, or sequentially planned after phacoemulsification; Exclusion criteria: Penetrating keratoplasty, prior intraocular surgery, and unspecified keratoplasty type; the denominators include the total cohort, encompassing both operated and unoperated cases, after excluding those that meet the exclusion criteria.

|  | **Number of Individuals** | **CTG18.1 Exp-** | **CTG18.1 Exp+** | | | **Biallelic:monoallelic ratio** | **Expected biallelic expanded cases^c^** |
| --- | --- | --- | --- | --- | --- | --- | --- |
|  |  |  | **Cases with ≥1 expanded allele** | **Monoallelic expanded cases** | **Biallelic expanded cases** |  |  |
| FECD cases of European Ancestry | 829 | 160 | 669 | 623 | 46 | 1:14 | 7 |
| Ethnicity-matched Controls^a^ | 550 | 527 | 23 | 23 | 0 | 1:94^b^ | <1 |

**eTable 4. Summary of CTG18.1 expansion status within Fuchs endothelial corneal dystrophy (FECD) probands of European ancestry and ethnicity-matched controls, where the derived allele frequency was used for expected and observed homozygous:heterzygous ratio calculation.** Exp+, CTG18.1 expansion positive allele defined as ≥50 CTG repeats; Exp-, CTG18.1 expansion positive allele defined as <50 CTG repeats. ^a^Data presented from Zarouchlioti et al. 2018.^1^ ^b^Control ratio under Hardy-Weinberg equilibrium that was confirmed in the control group (P=.617). ^c^Expected number of biallelic expanded cases based on control biallelic:monoallelic ratio and observed monoallelic expanded cases.

| **Model – single longest CTG18.1 allele measured per individual in all Exp+ patients** | **Age at first keratoplasty** | | | | |
| --- | --- | --- | --- | --- | --- |
| **Variables** | **Number of cases** | **Adjusted R^2^** | **Regression coefficient** | **95% CI** | ***P* value** |
| CTG18.1 repeat length of the largest allele | 308 | 0.013 | -0.087 | -0.162 to -0.012 | 0.024 |

**eTable 5. Linear regression models analyzing the relationship between CTG18.1 repeat length of the largest allele and age at first keratoplasty.** Exp+, CTG18.1 expansion positive allele defined as ≥50 CTG repeats.

| **Gene**  **CEC TPM** | **Transcript**  **(ENST00000-)** | **Patient ID** | **Variant** | **CDS** | **Protein** | **CADD** | **gnomAD (v3.1.2)** | | **Age at first keratoplasty (Years)** | **Sex** | **Ancestry^a^** | **Family history** | **Site** | **Previously reported** |
| --- | --- | --- | --- | --- | --- | --- | --- | --- | --- | --- | --- | --- | --- | --- |
|  |  |  |  |  |  |  | **Total** | **Max (Population)** |  |  |  |  |  |  |
| *AGBL1*  TPM = 0 | 614907.3 | P824 | chr15-86247772-A-T | c.628A>T | p.(Ile210Phe) | 18.3 | - | - | 68 | F | EUR | Unknown | MEH | No |
|  |  | P371 | chr15-86295271-T-C | c.2237T>C | p.(Leu746Pro) | 25.2 | - | - | 71 | F | EUR | Unknown | MEH | No |
|  |  | P864 | chr15-86674435-C-T | c.3157C>T | p.(Arg1053Trp) | 36 | 0.001952  297/152158 | 0.008353 (ASJ)  29/3472 | NS | M | EUR | Unknown | MEH | No |
|  |  | P759 | chr15-86907208-C-T | c.3280C>T | p.(Arg1094Trp) | 16.8 | 0.001007  153/152002 | 0.003020 (AFR)  125/41394 | 45 | F | AFR | Unknown | MEH | No |
| *LOXHD1*  TPM = 0.02 | 642948.1 | P2185 | chr18-46610832-T-G | c.703A>C | p.(Lys235Gln) | 26.0 | 0.00000656  1/152220 | 0.0000147  1/68030 | NS | M | EUR | No | GUH | No |
|  |  | P327 | chr18-46592017-G-A | c.1570C>T | p.(Arg524Cys) | 30 | 0.002833  431/152158 | 0.008903 (AMR)  136/15276 | 68 | F | EUR | Unknown | MEH | No |
|  |  | P354 | chr18-46592017-G-A | c.1570C>T | p.(Arg524Cys) | 30 | 0.002833  431/152158 | 0.008903 (AMR)  136/15276 | 73 | F | EUR | Unknown | MEH | No |
|  |  | P722 | chr18-46541815-G-A | c.3874C>T | p.(Leu1292Phe) | 25.7 | 0.0003088  47/152222 | 0.001223 (FIN)  13/10626 | 74 | M | EUR | Unknown | MEH | No |
|  |  | P2180 | chr18-46541787-A-C | c.3902T>G | p.(Leu1301Arg) | 16.2 | 0.00000657  1/152206 | 0.00006542 (AMR)  1/15286 | NS | F | EUR | No | GUH | No |
|  |  | P759 | chr18-46533293-C-T | c.4244G>A | p.(Arg1415Gln) | 27.3 | 0.0006768  103/152190 | 0.002293 (AFR)  95/41436 | 45 | F | AFR | Unknown | MEH | No |
|  |  | P523 | chr18-46529227-G-A | c.4480C>T | p.(Arg1494Ter) | 42 | 0.0006249  95/152026 | 0.001152 (ASJ)  4/3472 | 74 | F | EUR | Unknown | MEH | No |
|  |  | P399 | chr18-46522163-G-A | c.5023C>T | p.(Arg1675Cys) | 19.2 | 0.001512  230/152102 | 0.006743 (AMR)  103/15276 | 57 | F | AFR | Unknown | MEH | No |
|  |  | P788 | chr18-46505914-G-T | c.5802C>A | p.(Asn1934Lys) | 21.3 | 0.003082  469/152198 | 0.005624 (AMR)  86/15292 | 77 | F | EUR | Unknown | MEH | No |
|  |  | P522 | chr18-46477568-G-A | c.6726C>T | p.(Thr2242Thr) | 17.6 | 0.00002627  4/152258 | 0.00004823 (AFR)  2/41472 | 66 | F | EUR | Unknown | MEH | No |
|  |  | P737 | chr18-46477553-G-A | c.6741C>T | p.(Ala2247Ala) | 18.9 | 0.003061  466/152228 | 0.00463 (NFE)  315/68032 | 76 | F | NA | Unknown | MEH | No |

**eTable 6. Summary of rare variants identified in *LOXHD1* and *AGBL1* from 128 FECD CTG18.1 Exp- probands analysed by exome sequencing.** CEC, corneal endothelial cells; TPM, transcript per million; CADD, Combined Annotation Dependent Depletion; MAF, minor allele frequency; EUR, European; AFR, African American/African; FIN, Finnish; NFE, non-Finnish European, ASJ, Ashkenazi Jews; M, male; F, female; NS, no surgery; MEH, Moorfields Eye Hospital London; GUH, General University Hospital Prague.  ^a^FRAPOSA predicted ancestry.

**Online-Only References**

1. Zarouchlioti C, Sanchez-Pintado B, Hafford Tear NJ, et al. Antisense Therapy for a Common Corneal Dystrophy Ameliorates TCF4 Repeat Expansion-Mediated Toxicity. *Am J Hum Genet*. 2018;102(4):528-539.
